# Lessons Learned Developing a Diagnostic Tool for HIV-Associated Dementia Feasible to Implement in Resource-Limited Settings: Pilot Testing in Kenya

**DOI:** 10.1371/journal.pone.0032898

**Published:** 2012-03-07

**Authors:** Judith Kwasa, Deanna Cettomai, Edwin Lwanya, Dennis Osiemo, Patrick Oyaro, Gretchen L. Birbeck, Richard W. Price, Elizabeth A. Bukusi, Craig R. Cohen, Ana-Claire L. Meyer

**Affiliations:** 1 Center for Microbiology Research Kenya Medical Research Institute, Nairobi, Kenya; 2 Department of Neurology, University of California San Francisco, San Francisco, California, United States of America; 3 Michigan State University, East Lansing, Michigan, United States of America; 4 Department of Obstetrics, Gynecology and Reproductive Sciences, University of California San Francisco, San Francisco, California, United States of America; University of Nebraska Medical Center, United States of America

## Abstract

**Objective:**

To conduct a preliminary evaluation of the utility and reliability of a diagnostic tool for HIV-associated dementia (HAD) for use by primary health care workers (HCW) which would be feasible to implement in resource-limited settings.

**Background:**

In resource-limited settings, HAD is an indication for anti-retroviral therapy regardless of CD4 T-cell count. Anti-retroviral therapy, the treatment for HAD, is now increasingly available in resource-limited settings. Nonetheless, HAD remains under-diagnosed likely because of limited clinical expertise and availability of diagnostic tests. Thus, a simple diagnostic tool which is practical to implement in resource-limited settings is an urgent need.

**Methods:**

A convenience sample of 30 HIV-infected outpatients was enrolled in Western Kenya. We assessed the sensitivity and specificity of a diagnostic tool for HAD as administered by a primary HCW. This was compared to an expert clinical assessment which included examination by a physician, neuropsychological testing, and in selected cases, brain imaging. Agreement between HCW and an expert examiner on certain tool components was measured using Kappa statistic.

**Results:**

The sample was 57% male, mean age was 38.6 years, mean CD4 T-cell count was 323 cells/µL, and 54% had less than a secondary school education. Six (20%) of the subjects were diagnosed with HAD by expert clinical assessment. The diagnostic tool was 63% sensitive and 67% specific for HAD. Agreement between HCW and expert examiners was poor for many individual items of the diagnostic tool (K = .03–.65). This diagnostic tool had moderate sensitivity and specificity for HAD. However, reliability was poor, suggesting that substantial training and formal evaluations of training adequacy will be critical to enable HCW to reliably administer a brief diagnostic tool for HAD.

## Introduction

HIV-associated dementia (HAD) is an indication for initiating antiretroviral therapy regardless of CD4 T-cell count according to World Health Organization (WHO) guidelines (a WHO Stage IV diagnosis [1]. However, HAD is likely under-diagnosed in routine clinical practice in resource-limited settings [2]. Furthermore, HAD is still a clinically important disorder in resource-limited settings where many individuals present with advanced HIV disease. In research studies from sub-Saharan Africa, the prevalence of HAD ranges from 2.5%–54% [3]–[10]; these estimates vary widely likely due to differences in the sampled populations and methods for assessment of cognitive impairment. In contrast, the incidence of HAD in more developed regions has decreased dramatically since ART became widely available [11]–[14].

Focusing on the diagnosis of HAD as opposed to milder forms of HIV-associated neurocognitive disorders (HAND) is critical in settings where decisions about whether to initiate ART are frequently made based on WHO criteria alone and where ART treatment is available only to those with the greatest need—individuals with very low CD4 T-cell counts and/or WHO Stage III and IV diagnoses. Since HAD typically improves with ART [15]–[17], and availability of ART is increasing even in resource-limited settings, identification of individuals with HAD can be of great importance to improve health outcomes. However, the diagnosis of HAD remains a challenge in HIV outpatient primary care settings in resource-limited regions [2]. Potential reasons for this include: a lack of specialized personnel and diagnostic tests, and the inherent difficulties in making a clinical diagnosis of a complex disorder.

Several brief screening tests, like the International HIV Dementia Scale (IHDS), were developed to identify individuals with HAND in resource-limited settings. The IHDS has been demonstrated to be useful in detecting HAND in settings where screening is conducted by trained physicians and referral to a specialist is an option [9]. However, specialized personnel are rare in low income countries. In Kenya the median number of neurologists per 100,000 population is 0.03 as compared to 2.96 in high-income countries [18]; similarly, there are only 0.14 physicians per 1,000 population in Kenya as compared to the United States where the ratio is 2.56 [19].

In addition to highly specialized clinical expertise, in developed regions, the diagnosis of HAD often entails imaging of the brain, lumbar puncture, and in milder cases, neuropsychological testing. Diagnostic tests such as Computed Tomography (CT) of the head are widely available but unaffordable in Kenya, while magnetic resonance imaging (MRI) is even less affordable and only available in the capital city, Nairobi [20]. There are only two neuropsychologists in Kenya (both in private practice) [20]. Thus, neuropsychological testing is essentially unavailable, and, while some culturally specific normative data has recently become available for other sub-Saharan African populations, no published normative data exists for Kenyans [21], [22].

In summary, in resource-limited settings, non-physician health care workers (HCW) are the first and often the only health care provider available for a majority of HIV-infected individuals [23] and diagnostic tests are very limited. Therefore, our goal was to develop a simple diagnostic tool to identify individuals with HAD—individuals who should be started on ART regardless of their CD4 T-cell count. Furthermore, we felt it was important to develop a tool which would be feasible to implement in decentralized resource-limited settings. The diagnostic tool should not require referral to a specialist or additional diagnostic tests, should only require minimal additional training, and should be reliably administered by primary HCW. We created a diagnostic tool designed to guide a HCW through a clinical assessment for HAD; the tool includes a review of cognitive symptoms, a neurological examination to screen out individuals with focal neurological findings, cognitive tests to identify individuals with impairment, and an assessment of functional status. Here we report on challenges and lessons learned during pilot testing of this diagnostic tool for HAD designed for use by non-physician primary HCW in Nyanza province, Western Kenya.

## Methods

### Objectives

The objective of this study was to conduct a preliminary evaluation of the utility and reliability of a diagnostic tool for HAD designed for use by non-physician primary HCW.

### Participants

A convenience sample of 30 adults was recruited from an outpatient HIV care and treatment program run by Family AIDS Care and Education Services (FACES) in Kisumu, a city in Western Kenya, between September and October 2009. Potential participants were invited to participate when they presented for clinical care. One participant who had time to participate in the study (study activities took about 3 hours) was chosen each day by clinic staff and referred to study staff. In addition, patients with neurological findings were oversampled in order to assess the neurological examination portion of the tool—we enrolled any individual with neurological findings referred to us by study staff. Participants who could not speak English, Dholuo, or Kiswahili well enough to participate in the neuropsychological testing were excluded from the study.

### Procedures

A diagnostic tool was administered by a HCW. The results of this tool were compared to an expert clinical assessment performed by study staff which included: examination by a physician, a neuropsychological test battery administered by expert study staff, and computed tomography of the head when clinically indicated. The results of the expert clinical assessment were used to identify patients meeting a modification of the Frascati criteria for HAD [24]. The diagnostic tool was also administered by trained staff during the expert assessment to assess reliability.

### Diagnostic tool (administered by HCW)

A non-physician HCW administered the diagnostic tool (Appendix S1) which consisted of a brief history and neurological examination, cognitive assessment based on the International HIV Dementia Scale (IHDS) [9] and assessment of functional status. The HCW were blinded to the results of the expert clinical assessment performed by study staff. To assess reliability, all components of the cognitive testing portion of the diagnostic tool (C4–C8) were also administered by expert study staff. Since the tool was administered by HCW during the course of their usual clinical duties, the timing of tool administration was planned in such a way as to minimize impact to clinic flow. The expert clinical assessment was conducted after the HCW administered the diagnostic tool in 19/30 cases.

The eighteen non-physician HCW who participated in this study were either clinical officers or nurses on the staff of FACES. Clinical officers receive three to four years of post-secondary school education and are comparable to physician's assistants in the United States. Nurses receive two to four years of post-secondary school education. Since we sought to explore the utility of this diagnostic tool in a setting we felt mimicked “real-life” practice settings for primary HCW, we did not provide additional training specific to this tool to participating HCW. All the HCW who participated in the study have received HIV-specific training and ongoing mentorship through FACES. At the time of the study, one investigator (ACM) had been providing training and mentorship in HIV Neurology for six months at the study site and had conducted several trainings on the neurological examination to clinicians and nurses at our study site using a format quite similar to the neurological examination contained in the diagnostic tool. Most of the tests included in the cognitive assessment are part of the Kenyan National Guidelines, and in theory, all HCW had received training on their administration.

### Expert clinical assessment (administered by study team)

Each participant underwent a standardized clinical examination, a neuropsychological test battery, and computed tomography of the head when clinically indicated, and classification of dementia.

#### Examination

Each participant underwent a standardized examination which included: a detailed history; full medical examination; subjective and objective examination of mental state and cognition using the Patients Assessment of Own Functioning Inventory (PAOFI) [25] and the Mini-Mental State Examination [26]; detailed examination of all cranial nerves except the olfactory nerve; fundoscopy; motor examination and strength testing in 20 different muscle groups; examination of light touch, pinprick and temperature at 14 predefined areas, as well as assessment of distal joint position and vibration sense; six predefined tests of coordination and motor function; assessments of five deep tendon reflexes, the plantar reflex, four frontal release reflexes; gait exam with seven specific observations, and Romberg test. The two non-neurologists (DC and JK) who performed the neurologic examinations underwent extensive training including numerous full observed examinations by the principal investigator (AM). Computerized tomography (CT) of the brain was performed in four participants with likely HAD or focal findings on neurological exam to rule out a mass lesion, infarct, or other process which might mimic the clinical and neuropsychological findings of HAD. Several measures of functional status were used including the PAOFI, Activities of Daily Living [27], Instrumental Activities of Daily Living [28], and the Physical Function, Role Function, and Role limitations due to Physical Health of the Medical Outcomes Study [29]. Most of these have not been formally tested in a Kenyan population, so the answers to all these questions were integrated by the physician or neurologist in assigning a Karnovsky Performance Scale score [30] intended to represent limitations in function due only to cognitive impairment.

#### Neuropsychological test battery

The neuropsychological test battery was administered by expert study staff and assessed attention/working memory (Digit Span [31]), category fluency (Animals, First Names [32]) executive function (Block Design [31], Color Trails 2 [33]), speed of information processing (Digit Symbol [31], Color Trails 1 [33]), verbal and visual learning and memory (WHO/UCLA Auditory Verbal Learning Test [AVLT] [32], [33] and Revised Brief Visuospatial Memory Test [BVMT] [34]), and psychomotor speed (Grooved Pegboard [35], Finger Tapper [36], Timed Gait [37]). In addition, two established screening tests, the MMSE [26] and the International HIV Dementia Scale (IHDS) [9] were administered. As described previously, the MMSE was administered by the physician as part of the examination. Neuropsychological testing was performed in the patient's preferred language (either English, Kiswahili, or Dholuo). The study staff member administering neuropsychological tests was fluent in the preferred language.

As no published normative data was available from Kenyan or East African populations, normative values from general United States populations were used to convert raw scores to standardized scores (Z-scores) [31], [34]–[40]. The exception was for category fluency in which normative data from an African American U.S. population was used as data from a general population was not available [38]. Z-scores were calculated so that negative Z scores always represented worse performance on a test. A composite standardized score was generated by averaging the mean standardized score for each individual test.

#### Classification of Dementia

The Frascati criteria for HAD were modified slightly and implemented as follows [24]: for an individual to be classified with HAD, they had to have (1) neuropsychological test scores >2 standard deviations (SD) below the mean in at least two different cognitive domains, and (2) major functional decline due to cognitive impairment in the opinion of the examining physician. We omitted a second criterion of impairment on neuropsychological tests (a score >1 SD mean in one cognitive domain and a score of >2.5 SD below the mean in another) because we did not have a culturally appropriate normative sample and nearly all of our participants scored >1SD below the mean in at least one cognitive domain. For an assessment of the performance of neuropsychological tests we divided our sample into two additional categories. Individuals who did not meet the above criteria for abnormal neuropsychological tests were classified as normal. The remaining individuals who met the neuropsychological test criteria for cognitive impairment as described above did not have no major impairments in functional status were grouped and classified as Asymptomatic Neurocognitive Impairment (ANI)/Mild Neurocognitive Disorder (MND). Subjects were classified by a physician and a neurologist and final diagnoses were decided by consensus. The physician and neurologist were blinded to the results of the diagnostic tool administered by the HCW.

### Analytic Approach and Statistical Methods

Descriptive statistics were generated for demographic and clinical characteristics. Student's T-tests were used to compare means and Fisher's exact test to compare proportions between individuals with and without HAD. We also compared raw and standardized scores on neuropsychological tests across categories of HAND and between individuals with and without moderate to severe depression. Sensitivity, specificity, receiver operator characteristic (ROC) curves were generated for the diagnostic tool. Quadratic-weighted kappa statistics were generated for the overall score and individual items on the cognitive portion of the diagnostic tool (Section C4–C8) to compare agreement between the HCW and expert study staff.

Statistical analyses were performed using Stata10.0 (StataCorp, College Station, Texas). A significance level of p≤.05 was used.

### Ethics Statement

The study protocol was approved by the University of California San Francisco (UCSF) Committee on Human Research and the National Ethical Review Committee (ERC) of the Kenya Medical Research Institute (KEMRI). Written informed consent was obtained from each participant.

## Results

### Expert Clinical Assessment

The mean age of the sample was 39 years and 57% were male. The mean CD4 T-cell count was 323 cells/µL, 60% had WHO Stage 3 or 4 disease, and 50% had less than a secondary school education (Table 1). Nearly 23% (7/30) of participants had moderate or severe depression using the Patient Assessment of Health Questionnaire (PHQ9) [41]. In general, functional status measures demonstrated mild impairments. There were no significant differences in demographic, clinical, functional status or psychiatric characteristics between individuals with and without HAD though this should be interpreted with caution given small sample sizes.

**Table 1:** Baseline characteristics of study participants comparing individuals with and without HIV-associated dementia.

	Overall (n = 30)	No dementia (n = 24)	HIV-associated dementia (n = 6)
*Demographic Characteristics*			
Age *[mean (SD)]*	39 (10)	47 (6.8)	37 (10)
Male *[% (n)]*	57% (17)	50% (12)	83% (5)
*Marital Status [% (n)]*			
Never Married	13% (4)	17% (4)	0%
Married	63% (19)	54% (13)	83% (5)
Widowed	20% (6)	21% (5)	17% (1)
Divorced/separated	4% (1)	4% (1)	0%
*Highest level of education achieved [% (n)]*			
Less than primary	23% (7)	21% (5)	33% (2)
At least primary	27% (8)	29% (7)	17% (1)
Secondary	37% (11)	42% (10)	17% (1)
More than secondary	14% (4)	12% (2)	33% (2)
*Socio-economic status*			
Electricity in dwelling *[% (n)]*	37% (11)	38% (9)	33% (2)
Running water in dwelling *[% (n)]*	13% (4)	13% (3)	17% (1)
*Selected clinical characteristics*			
*WHO Stage currently [% (n)]*			
Stage 1	13% (4)	13% (3)	17% (1)
Stage 2	27% (8)	29% (7)	17% (1)
Stage 3	37% (11)	33% (8)	50% (3)
Stage 4	23% (7)	25% (6)	17 (1)
Nadir CD4 cell count *[mean (SD)]*	198 (150)	215 (157)	127 (103)
Current CD4 cell count *[mean (SD)]*	323 (211)	337 (227)	268 (134)
Moderate to severe depression *[%(n)]* *	23% (7)	21% (5)	33% (2)
*Functional Status Measures*			
Karnovsky Performance Scale *[mean (SD)]Range (0–100)*	92 (20)	92 (22)	91 (4.1)
Activities of Daily Living (ADL)*[mean (SD)] Range (0–4)*	4 (0)	4 (0)	4 (0)
Independent ADL *[mean (SD)] Range (0–8)*	7.8 (0.7)	7.8 (0.7)	8 (0)
Physical Function*[mean (SD)] Range (0–100)*	89(19)	97 (4)	87 (201)
Role Limitation due to Physical Function *[mean (SD)] Range (0–100)*	82 (5.1)	80 (31)	89 (16)
Patient assessment of own functioning inventory (PAOFI)† *[mean (SD)] Range (0–165)*	12 (14)	13 (15)	12 (5.9)

* *Score ≥10 on Patient Health Questionnaire (PHQ9);*

† *PAOFI- a score of 0 reflects no impairment.*

Among our pilot sample, 20% (6/30) were diagnosed with HAD based on the expert clinical assessment. An additional 33% (10/30) were classified as normal. The remaining 47% (14/30) individuals were grouped and classified as mild neurocognitive impairment. Among the individuals classified as normal, there were five individuals with abnormal neurological examinations potentially localizing to the central nervous system. Findings included: (1) unsteady gait and asymmetric ataxia (left worse than right); (2) multiple cranial neuropathies (III, IV, V, VI, VII, IX, X) with subtle left sided weakness and left cerebellar signs; (3) parkinsonism; (4) a large left retro-orbital mass, and (5) a history of right-sided weakness with no objective findings on examination. Of these individuals, three individuals were referred for computed tomography (CT) of the head. The CT findings were as follows: (1) Marked cerebellar volume loss including the middle cerebellar peduncle and pons but sparing the medullary pyramids; (2) periventricular hypothalamic lesion and marked edema, likely CNS lymphoma, (3) diffuse volume loss and small caudate heads. The individual with a left retro-orbital mass had a CT scan previously that demonstrated a sizeable mass in the retro-orbital space. A fourth individual who had markedly poor performance on neuropsychological tests and who was ultimately classified as having HAD, also underwent a CT scan which was normal.

#### Neuropsychological tests and HAND

**Figure 1:**
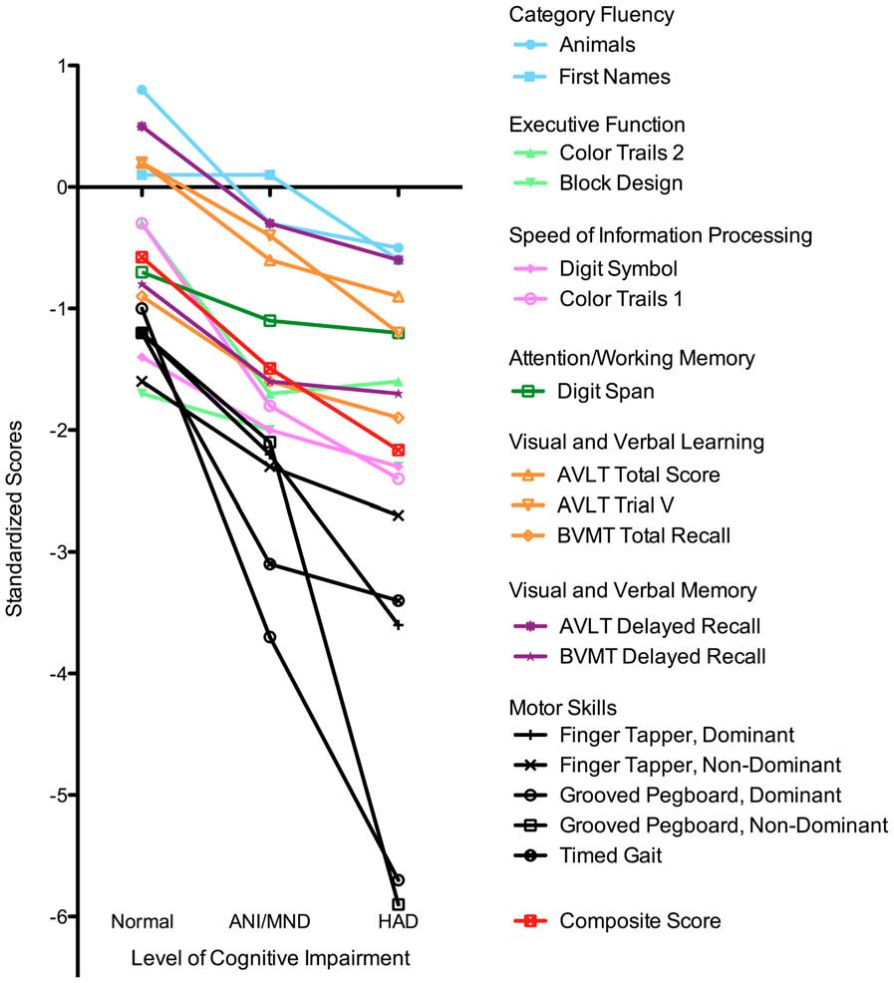
Comparison of mean standardized scores on individual neuropsychological tests comprising neuropsychological test battery and a composite measure by level of cognitive impairment among HIV-infected individuals.

We then reviewed the performance of our neuropsychological test battery, excluding the four individuals listed above with abnormal neurological examinations. As the level of cognitive impairment increased, mean raw scores worsened on nearly all of the individual neuropsychological tests chosen for this test battery (Table 2). Similarly, individuals with higher levels of cognitive impairment had worse performance on the MMSE and IHDS. The one exception was Category Fluency- First Names where mean raw scores among individuals with mild neurocognitive impairment were higher than scores among normal individuals. Mean standardized scores also worsened on all tests except Category Fluency-First Names as the level of cognitive impairment increased (Figure 1). The composite score, an average of the standardized scores for all the tests, decreased from -0.6 [SD: 0.76] among normal individuals to -2.16 [SD: 1.63] among individuals with HAD. The greatest magnitude differences on standardized scores between normal individuals and those with HAD were on the tests of Motor Skills and Color Trails 1. Of note, among individuals classified as normal, the mean standardized score was ≤−1 in Block Design, Digit Symbol, and all the tests of Motor Skills.

**Table 2:** Comparison of mean raw scores on individual tests comprising neuropsychological test battery by level of cognitive impairment among HIV-infected individuals.*

	Normal		ANI, MND		HAD	
	(n = 6)		(n = 14)		(n = 6)	
	Mean	SD	Mean	SD	Mean	SD
**Category Fluency**						
Animals	20.8	(5.5)	16.6	(3.8)	14.8	(4.1)
First Names	23	(4.3)	23.1	(5.4)	19.3	(5.5)
**Executive Function**						
Color Trails 2†	118.8	(28.9)	159.2	(46.5)	169.5	(42.9)
Block Design	16.2	(7.3)	11.8	(2.5)	7.3	(3.3)
**Speed of Information Processing**						
Digit Symbol	51.8	(12)	34.5	(10.9)	23	(14.0)
Color Trails 1†	51.2	(9.3)	76.3	(29.0)	99.3	(28.9)
**Attention/Working Memory**						
Digit Span	13.8	(2.1)	12.2	(3.2)	11.2	(4.7)
**Visual and Verbal Learning**						
AVLT Total Score	52.8	(3.2)	45.1	(8.0)	41.3	(6.7)
AVLT Trial V	12.5	(1.4)	11.1	(2.0)	9.3	(2.7)
BVMT Total Recall	21.8	(4.8)	17.4	(8.2)	13.2	(6.9)
**Visual and Verbal Memory**						
AVLT Delayed Recall	12.2	(1.7)	9.4	(2.9)	7.7	(2.3)
BVMT Delayed Recall	8.8	(1.8)	6.3	(3.6)	5.7	(2.9)
**Motor Skills**						
Finger Tapper (D)	44.7	(5.7)	38.3	(5.7)	34.4	(10.1)
Finger Tapper (ND)	38.2	(6.4)	33.8	(4.1)	33.5	(7.7)
Grooved Pegboard (D)†	71.2	(10.3)	91.7	(25.6)	111.3	(27.8)
Grooved Pegboard (ND)†	82.5	(12.3)	93.9	(14.5)	132.5	(30.2)
Timed Gait†	11	(1.5)	13.3	(2.3)	13.5	(2.8)
**Batteries**						
International HIV Dementia Scale	11	(1.7)	9.9	(1.9)	8.5	(1.8)
Mini-Mental State Examination	27.5	(1.9)	27.1	(2,1)	26.5	(2.2)

* Four individuals with non-HIV related neurologic diagnoses that affected performance on neuropsychological tests were dropped from this analysis.

† Higher scores indicate worse performance. Abbreviations: ANI: Asymptomatic Neurocognitive Impairment; D = Dominant; HAD: HIV-associated dementia; MND: Mild Neurocognitive Disorder; ND = Non-dominant.

#### Neuropsychological tests and depression

Individuals with moderate to severe depression on the PHQ-9 instrument were not more likely to have a score of ≤22 on our novel diagnostic tool using Fisher's exact (.39). They were more likely to have a score of ≤10 on the IHDS using Fisher's exact (.02). In general, individuals with moderate to severe depression had significantly lower mean standardized scores on all the neuropsychological tests from the Psychomotor domain (Grooved Pegboard (Dom/ND), Finger Tapper (Dom/ND), Timed Gait) as well as the verbal learning and memory domain (AVLT Total Score, AVLT Score Trial V, AVLT DR) (data not shown). The remainder of the tests did not demonstrate significant differences in mean standardized scores between with individuals with and without moderate to severe depression: Category fluency (animals and names), Color Trails 1 & 2, Block Design, Digit Symbol, Digit Span, BVMT, BVMT-DR (data not shown).

### Novel Diagnostic tool and HAD

Among this sample, the diagnostic tool determined that 53% (16/30) of our sample had HAD. An ROC curve was generated to assess the utility of the diagnostic tool in identifying individuals with HAD. A cut-off score of ≤22 (maximum = 26) resulted in a sensitivity of 63%, specificity of 67% and accuracy of 63%. Kappa statistics were used to evaluate agreement between the neuropsychological tests administered by a HCW as compared to expert study staff in Section C (cognitive portion) of the diagnostic tool. An expert examiner administered the cognitive components of the diagnostic tool after the HCW in 19/30 cases; in the remaining 11 cases, the expert examiner administered the diagnostic tool first. As shown in Table 3, moderate agreement was observed for C6: Serial Operations and C7: Category Fluency. Fair agreement was observed for C8: Recall and the total neuropsychological test score (C score). There was poor agreement for C4: Finger tapping and C5: Luria sequence. Despite poor agreement on 2 of 3 items taken from the IHDS, there was fair agreement on the overall score of the IHDS. Similarly there was poor agreement between Section B (neurological examination portion) of the diagnostic tool and the neurological examination conducted by the study physician; four individuals found to have an abnormal neurological exam by the study physician were not similarly classified when examined by the HCW administering the diagnostic tool.

**Table 3:** Agreement between non-physician health care worker and a expert examiner on cognitive tests in the diagnostic tool.

Neuropsychological test Items from diagnostic tool (Section C)	Quadratic weighted Kappa
C4: Finger Tapping	0.03
C5: Luria sequence	0.05
C6: Serial Operations	0.47
C7: Category fluency (animals)	0.65
C8: Recall	0.25
***C score (Sum of C1–C8)***	0.32
***International HIV Dementia Scale total score (Sum of C4, C5,C8)***	0.26

## Discussion

### Expert Clinical Assessment

This convenience sample of individuals presenting for routine clinical care in Kenya was comparable to larger community-based samples from sub-Saharan Africa [42]–[44]. In addition, during the month that we were recruiting for this study, we encountered several individuals with severe neurological disorders—highlighting the importance of improving neurological training for primary HIV healthcare workers in resource-limited settings.

#### Neuropsychological Test Battery and HAND

Mean raw and standardized scores (Z-scores) on many tests in our neuropsychological test battery demonstrated large magnitude differences between individuals with and without HAND. Our results demonstrated the typical pattern of HIV associated cognitive impairment with one notable exception. Our results were similar to prior studies in that we observed large differences in scores in the domains of executive function (Block Design, Color Trails 2,) speed of information processing (Digit Symbol, Color Trails), and motor/psychomotor function (Grooved Pegboard, Finger Tapper, and Timed Gait) [45]–[49].

In contrast to previous studies, tests of motor or psychomotor performance in the non-dominant hand did not demonstrate larger magnitude differences in mean raw or standardized scores and thus may not be more sensitive and reliable in diagnosing HAND [10], [22]. This may be related to the small sample size or cultural differences in interpreting test instructions. For example, we noted that many study subjects did not work as quickly as they could after being given the instruction “as fast as you can” and that additional explanation was necessary to elicit maximal effort on timed tests. These differences warrant further exploration with a larger sample size and culturally specific normative data before any conclusions can be drawn.

We found large differences in mean raw and standardized scores for AVLT and BVMT-R among individuals with and without HAND although difficulty with verbal learning and memory are thought to be less characteristic of HAND [50]. These findings were comparable to those reported from a Ugandan study which used AVLT as part of their test battery [22]. Whether this represents a different pattern of cognitive deficits unique to sub-Saharan Africa, is related to literacy levels, or is a function of overlapping cognitive domains (e.g. low scores on AVLT could be caused by poor attention) remains to be seen.

One important finding is that among individuals classified as normal, performance on several tests was substantially lower than our U.S. normative population. Mean standardized scores on Block Design, Digit Symbol, and all the tests of motor skills were less than or equal to one standard deviation below the normative mean. Strictly applying the AAN Frascati criteria [24] could result in a diagnosis of mild cognitive impairment at this level of performance, again highlighting the need for a culturally appropriate normative sample.

### Novel Diagnostic Tool and HAD

As administered in this pilot sample, the novel diagnostic tool had lower sensitivity and specificity than studies using similar tools—the IHDS in Uganda [9] and a culturally adapted version of the MMSE in Zambia [3]. This study differed from those studies in several important ways. First, in this study, a non-physician HCW administered the diagnostic tool without additional training while in the Ugandan study, a highly trained physician administered the IHDS [9]. Second, we introduced a novel assessment tool while in the Zambian study, the Zambian version of the MMSE was familiar to the primary HCW from their clinical training [3]. In the same study, the HIV Dementia Scale (HDS) [51] was also administered by trained HCW. Even with training, frequent errors were reported in the test which may have been due in part to poor administration by HCW [42].

As described in the methods section, the HCW participating in this study had received some general training in the neurological examination and in HIV care according to Kenya National Guidelines which includes many of the cognitive tests contained in the diagnostic tool. In this study, we chose not to provide additional training specific to the tool for two reasons: (1) to assess training needs for this tool, and (2) to assess the feasibility of implementation in a decentralized health care system where most care is provided by non-physician HCW and wide-scale comprehensive training is not feasible. However, the lack of specific training likely contributed to the poor sensitivity of the tool. For example, there was poor agreement between the non-physician HCW and the study physician on the neurological examination. In addition there was poor agreement on many of the cognitive items comprising the diagnostic tool between HCW and expert study staff.

These findings suggest that substantial training and assessments of inter-rater agreement between physicians and non-physician HCW will be critical prior to the use of this diagnostic tool in routine clinical settings. Providing ongoing training is a significant challenge in resource limited settings where staff turn-over is high. Alternatively, choosing an assessment tool, or components of an assessment tool, that are simpler to administer or more familiar to primary HCW may improve diagnostic utility in routine clinical settings. To maximize the applicability of HAD screening tools in a decentralized system one may have to trade diagnostic sophistication and precision—measuring the cognitive domains most affected in HAD—to develop a tool that is easy to teach and easy to use.

Another potential reason for the lower utility of the diagnostic tool is inclusion of individuals with co-morbid depression who may have been misclassified as having HAD because of poor performance on cognitive assessments [52]. Furthermore, the symptom (Section C, items 1 and 2) and functional status questions (Section D) on the tool showed little variation and likely added little to the ability of the tool to discriminate between those with and without HAD (data not shown). Finally, although the patient populations were demographically similar, cultural differences between our study site in Kenya and those in Zambia and Uganda may have contributed to the lower utility in our population.

### Strengths and Limitations

A strength of our study is that it was performed in an outpatient HIV care setting typical of others in sub-Saharan Africa. In addition, the diagnostic tool was administered by non-physician HCW which improves the generalizability of our results. However, this study was limited by the small sample size and the lack of training for non-physician HCW administering the tool. Also, the neuropsychological test instructions were not standardized but were interpreted by study staff fluent in those languages. In addition, the expert clinical assessment by study staff did not include a lumbar puncture to evaluate for other infections of the central nervous system or MRI to look for other focal lesions of the brain. In place of these procedures, which are not available in our setting, we performed a detailed medical and neurological examination. We also performed CT of the head in individuals with HAD or an abnormal neurological examination that localized to the central nervous system.

It is important to note that a major limitation of this study was the lack of Kenyan normative data. Since performance on many neuropsychological tests is sensitive to cultural, linguistic, educational, gender and age differences, it is not known if (or to what extent) the tests scores of an HIV uninfected Kenyan normal population would differ from this HIV infected population. Our study demonstrates that many normal individuals receive scores in the impaired range on several of the neuropsychological tests, suggesting that normative data from the U.S. is not appropriate, and highlighting the need for culturally appropriate normative data.

### Conclusion

Pilot testing suggests that substantial training and formal evaluations of training adequacy will be critical to enable primary HCW to reliably administer a brief diagnostic tool for HAD that contains cognitive tests and a neurological examination. Alternatively, selecting the simpler or more familiar components from more sophisticated screening or diagnostic tools may improve the diagnostic utility in routine clinical settings in a decentralized system of care. Finally, normal individuals frequently have standardized scores in the impaired range using our neuropsychological test battery. Thus, culturally specific normative values for neuropsychological tests are critical prior to widespread clinical or research use for the diagnosis of HIV-associated neurocognitive disorders.

## Supporting Information

Appendix S1 **HIV Dementia Diagnostic Tool.**
(DOC)
