# Supplementary material for: Lessons Learned Developing a Diagnostic Tool for HIV-Associated Dementia Feasible to Implement in Resource-Limited Settings: Pilot Testing in Kenya
Source: PLoS One. 2012 Mar 7;7(3):e32898. doi: 10.1371/journal.pone.0032898 (PMC3296754; doi:10.1371/journal.pone.0032898)
Supplement: Appendix S1 — HIV Dementia Diagnostic Tool. (DOC) [file pone.0032898.s001.doc]

| **HIV DEMENTIA DIAGNOSTIC TOOL** | | | | | | | |
| --- | --- | --- | --- | --- | --- | --- | --- |
|  | | | | | | | |
| ***Section A: Medical Assessment*** | | | | | | | |
| A1. | Does the patient have a fever >37.5 degrees Celsius? | | | | | | |
| **If YES, then stop assessment and repeat when afebrile.** | | | | | | | |
|  |  |  | |  | | | |
| ***Section B: Neurological Examination*** | | | | | | | |
|  | *Examination* | | Score | | Scoring Instructions | | |
| B1. | Is the patient alert and keenly responsive? | |  | | 1= Not alert  0= alert, keenly responsive | | |
| B2. | Is patient able to speak normally, make conversation and answer questions? | |  | | 1= difficulty expressing themselves, is unable to speak in complete sentences or makes errors using words.  0= speak normally, make conversation, answer questions | | |
| B3. | Is patient’s speech easily understood? | |  | | 1= slurs words or has difficult to understand speech  0= easily understood | | |
| B4. | Place two fingers of examiner’s hand in patients’ right hand and say “Squeeze my hand” | |  | | 1= unable to follow command  0= able to follow command | | |
| B5. | “Without moving your head, use your eyes to follow my finger.” Move finger to left and right and examine eye movements | |  | | 1= one or both eyes cannot move completely to left or right.  0= both eyes move completely to left and right | | |
| B6. | Ask patient to 1) show teeth,  2) raise eyebrows, then 3) close eyes. | |  | | 1= flattened nasolabial fold, asymmetric smile or paralysis of one side of face.  0= symmetric full facial movements | | |
| B7. | *Ask patient to extend arms straight forward at 90 degrees while in seated position with palms down and hold arms up for 10 seconds🡺🡺* | | | | | | 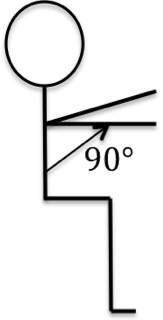 |
|  | a. Able to hold right arm up for 10 seconds. | |  | | 1= unable to hold arm at 90 degrees for full 10 seconds  0= able to perform task | |  |
|  | b. Able to hold left arm up for 10 seconds | |  | | 1= unable to hold arm at 90 degrees for full 10 seconds  0= able to perform task | |  |
| B8. | Ask patient to touch their nose, then the examiners finger. Repeat 3 times on each side | |  | | 1= movement is wobbly, irregular or jerky.  0= smooth accurate movements | | |
| B9. | *Ask patient to lie flat on back and raise each leg completely extended to 30 degrees and hold for 5 seconds. Test each leg one at a time.🡺🡺* | | | | | 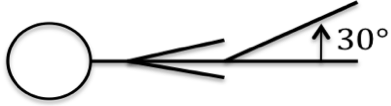 | |
|  | a. Able to hold right leg at 30 degrees for 5 seconds | |  | | 1= unable to hold leg at 30 degrees for full 5 seconds  0= able to perform task | | |
|  | b. Able to hold left leg at 30 degrees for 5 seconds | |  | | 1= unable to hold leg at 30 degrees for full 5 seconds  0= able to perform task | | |
|  | **Total Score (Add B1-9)** | |  | |  | | |
| **If score is > 3 on questions B1-11 stop assessment and refer to a physician for further evaluation.** | | | | | | | |

| ***Section C: History and Neuropsychological Testing*** | | | | |
| --- | --- | --- | --- | --- |
|  | *History* | | Score | Scoring Instructions |
| C1. | Have you noticed a decrease in your concentration, memory, or speed of thought which lasted for more than six months? | |  | 1= No  0= Yes |
| C2. | Have you experienced memory loss and/or confusion which lasted for more than six months? | |  | 1= No  0= Yes |
| ***C3. Give four words to recall (dog, hat, bean, red)*** | | | | |
|  | Ask the patient which hand they write or eat with. This is the dominant hand. | |  | L=left  R=right |
| C4. | Ask patient to tap the first 2 fingers of the non-dominant hand as wide and quickly as possible | 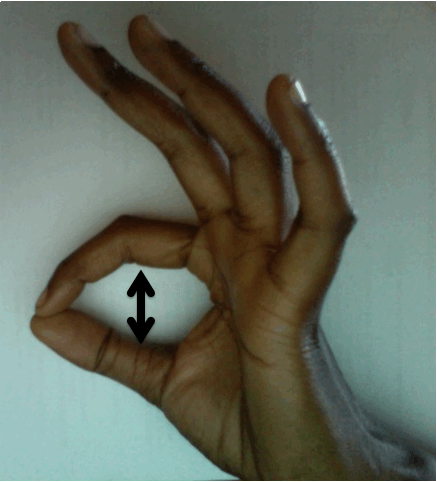 |  | 4= >15 in 5 seconds  3= 11-14 in 5 seconds  2= 7-10 in 5 seconds  1= 3-6 in 5 seconds  0= 0-2 in 5 seconds |
| C5. | Ask patient to perform the following sequence of **3** movements with the non-dominant hand as quickly as possible. Demonstrate and have the patient perform twice for practice: | |  | 4= 4 sequences in 10 seconds  3= 3 sequences in 10 seconds  2= 2 sequences in 10 seconds  1= 1 sequence in 10 seconds  0= Unable to perform |
|  | 1) Clench the hand in a fist and tap the side of the 5^th^ digit on the table | 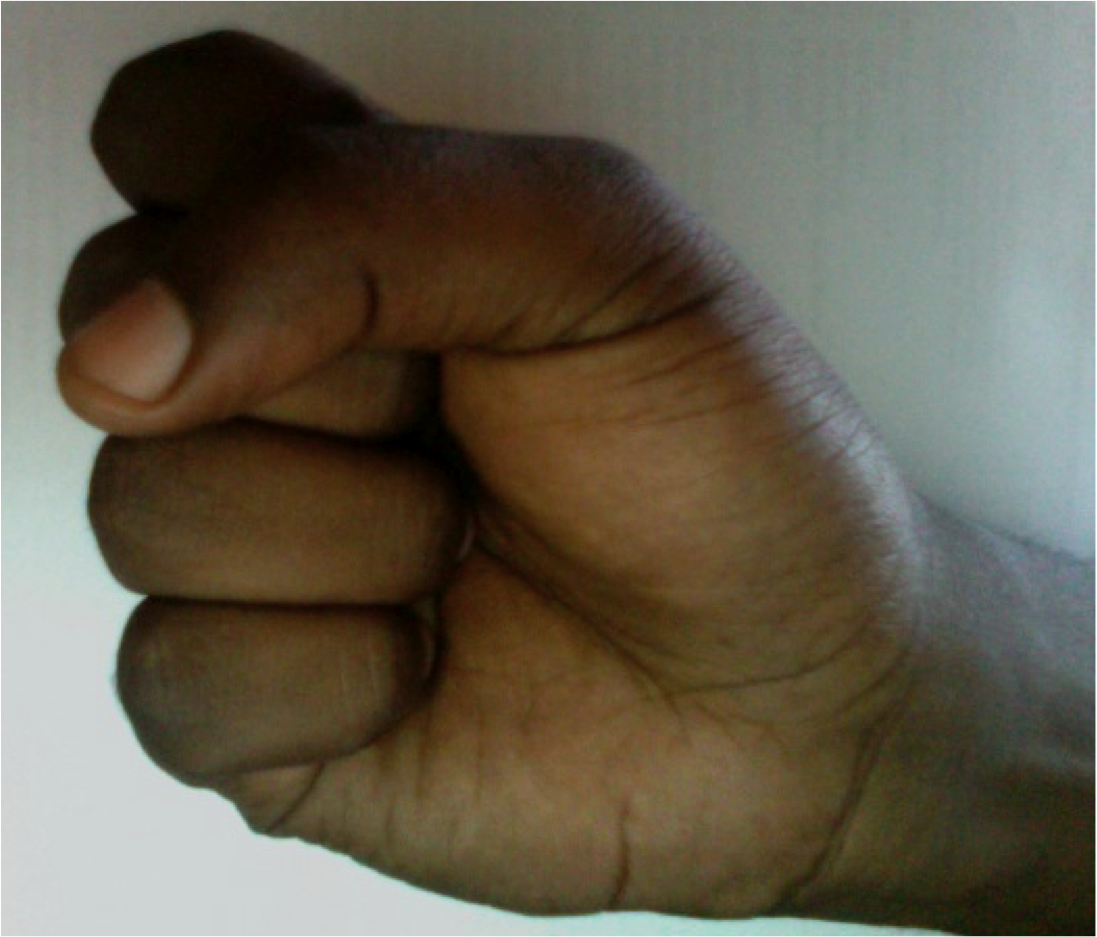 |  |  |
|  | 2) Put hand flat with palm down and tap on table | 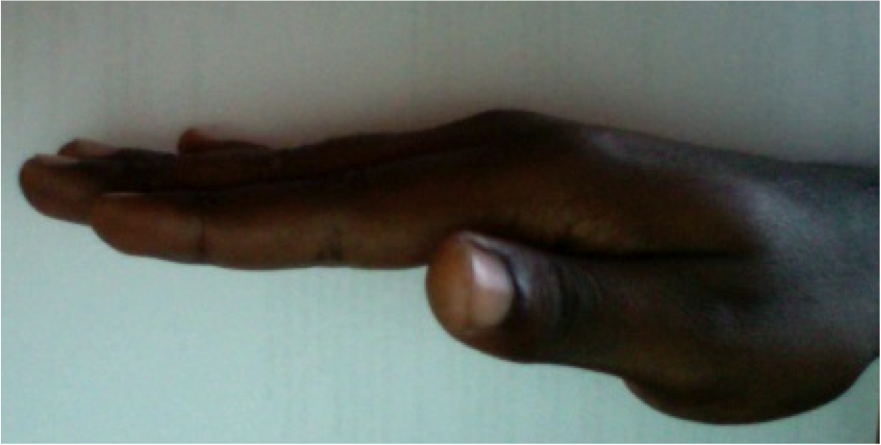 |  |  |
|  | 3) Put hand perpendicular and tap the table with the side of the 5^th^ digit | 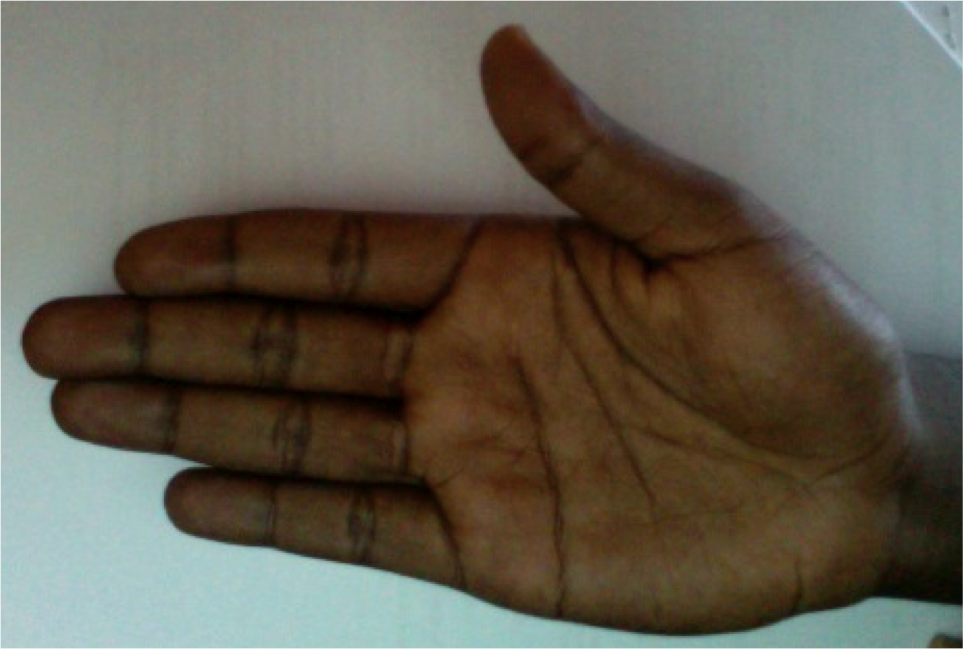 |  |  |
| C6. | Ask patient to count backward from 20. | |  | 2= Completes in <30 seconds  1= Completes in >30 seconds  0= Unable to complete |
| C7. | Name all the animals you can think of in 60 seconds | |  | 4= >20 animals  3= 15-20 animals  2= 8-14 animals  1= 0-7 animals  0= Unable to perform |
| C8. | Ask the patient to recall the four words. For words not recalled prompt with a sematic clue as follows: animal (dog); piece of clothing (hat); food (bean); colour (red) | |  | Give 1 point for every word spontaneously recalled.  Give 0.5 points for each correct answer after prompting.  Maximum=4 points |
|  | **Total Score (Add C1-C8)** | |  | Maximum 20 points |

| ***Section D. Functional Status*** | | | | |
| --- | --- | --- | --- | --- |
|  | | | | |
| When you compare what you can do **now** with what you could do before you were diagnosed with HIV, how much do problems with thinking or slowness of movement interfere with: | | | | |
|  |  | Does not interfere at all | Interferes a little bit | Interferes a lot |
| D1. | Your normal work (including both work outside the home and housework)? | 2 | 1 | 0 |
| D2. | Normal social activities with family, friends, neighbors, or groups such as religious worship? | 2 | 1 | 0 |
| D3. | Your ability to walk or move about | 2 | 1 | 0 |
|  |  |  |  |  |
|  | **Total Score (Add D1-D3)** |  | Maximum 6 points | |

| **THINK Dementia Diagnostic Score**  ***(Add Total Score from Sections C and D)***  ***(Maximum 26 points)*** |  |
| --- | --- |
